# Intrauterine resuscitation during the second stage of term labour by maternal hyperoxygenation versus conventional care: study protocol for a randomised controlled trial (INTEREST O2)

**DOI:** 10.1186/s13063-018-2567-x

**Published:** 2018-03-23

**Authors:** Lauren M. Bullens, Alexandra D. J. Hulsenboom, Suzanne Moors, Rohan Joshi, Pieter J. van Runnard Heimel, M. Beatrijs van der Hout-van der Jagt, Edwin R. van den Heuvel, S. Guid Oei

**Affiliations:** 1 Department of Obstetrics and Gynaecology, Máxima Medical Centre, PO Box 7777, 5500 MB Veldhoven, The Netherlands; 2 Department of Electrical Engineering, Eindhoven University of Technology, PO Box 513, 5600 MB Eindhoven, The Netherlands; 3 Department of Clinical Physics, Máxima Medical Centre, PO Box 7777, 5500 MB Veldhoven, The Netherlands; 4 Department of Industrial Design, Eindhoven University of Technology, PO Box 513, 5600 MB Eindhoven, The Netherlands; 5 Department of Mathematics and Computer Science, Eindhoven University of Technology, PO Box 513, 5600 MB Eindhoven, The Netherlands

**Keywords:** Foetal distress, Foetal heart rate, Cardiotocogram, Intrauterine resuscitation, Maternal hyperoxygenation, Neonatal outcome, Free oxygen radicals, Randomised controlled trial

## Abstract

**Background:**

Perinatal asphyxia is, even in developed countries, one the major causes of neonatal morbidity and mortality. Therefore, if foetal distress during labour is suspected, one should try to restore foetal oxygen levels or aim for immediate delivery. However, studies on the effect of intrauterine resuscitation during labour are scarce. We designed a randomised controlled trial to investigate the effect of maternal hyperoxygenation on the foetal condition. In this study, maternal hyperoxygenation is induced for the treatment of foetal distress during the second stage of term labour.

**Methods/design:**

This study is a single-centre randomised controlled trial being performed in a tertiary hospital in The Netherlands. From among cases of a suboptimal or abnormal foetal heart rate pattern during the second stage of term labour, a total of 116 patients will be randomised to the control group, where normal care is provided, or to the intervention group, where before normal care 100% oxygen is supplied to the mother by a non-rebreathing mask until delivery. The primary outcome is change in foetal heart rate pattern. Secondary outcomes are Apgar score, mode of delivery, admission to the neonatal intensive care unit and maternal side effects. In addition, blood gas values and malondialdehyde are determined in umbilical cord blood.

**Discussion:**

This study will be the first randomised controlled trial to investigate the effect of maternal hyperoxygenation for foetal distress during labour. This intervention should be recommended only as a treatment for intrapartum foetal distress, when improvement of the foetal condition is likely and outweighs maternal and neonatal side effects.

**Trial registration:**

EudraCT, 2015-001654-15; registered on 3 April 2015. Dutch Trial Register, NTR5461; registered on 20 October 2015.

**Electronic supplementary material:**

The online version of this article (10.1186/s13063-018-2567-x) contains supplementary material, which is available to authorized users.

## Background

Labour contractions cause alterations in intrauterine pressure and can thereby affect uterine and umbilical blood flow [1–5]. These fluctuations in blood flow towards the foetus can negatively influence oxygen flow and blood pressure [1–5]. Through chemo- and baroreceptor responses, these changes in foetal oxygenation and blood pressure affect foetal heart rate (FHR) [1, 2, 6, 7]. Hence, non-reassuring FHR patterns such as FHR decelerations may be a sign of foetal hypoxia [8–10]. Prolonged foetal hypoxia may lead to an increased risk of foetal morbidity, including renal insufficiency, pulmonary hypertension, necrotising enterocolitis and hypoxic–ischemic encephalopathy and foetal death [11, 12]. A prospective cohort study of term neonates in 2010 showed that 48% of admissions of these neonates to neonatal intensive care units (NICUs) were related to perinatal asphyxia (defined by the authors as a 5-minute Apgar score < 7). The neonatal mortality rate was 8% in this study, the largest proportion of which (71%, *n* = 12 of 17) was related to asphyxia [13].

Methods to directly measure foetal oxygenation during labour are unavailable, whereas methods for the continuous intrapartum monitoring of pH, oxygen saturation (SpO_2_), partial carbon dioxide pressure (pCO_2_) and partial oxygen pressure (pO_2_) are not yet suitable for clinical practice [14–16]. Therefore, the cardiotocogram (CTG), with occasional foetal scalp blood sampling (FSBS), is still the method of first choice to estimate foetal well-being during labour. The CTG has very good specificity but poor sensitivity for foetal well-being [17]. In other words, if the FHR pattern is reassuring, the foetus is very likely to be well-oxygenated. However, when FHR patterns are non-reassuring, the foetal condition is unclear, and foetal distress cannot be ruled out.

Instead of aiming for immediate delivery in the presence of suspected foetal distress, one may try to improve foetal oxygenation to avoid an invasive intervention. Several intrauterine resuscitation techniques are used in clinical practice and have been described in the literature [18, 19]. However, robust evidence regarding their effect on neonatal outcome is limited [18]. One of the interventions that still raises discussion is the administration of additional oxygen to the mother to treat foetal distress during labour [18, 20–23].

### Summary of findings from clinical studies

In the past five decades, researchers in several studies have investigated the effect of maternal hyperoxygenation on maternal and foetal oxygenation. Indeed, they found increasing maternal pO_2_ [24] and foetal SpO_2_ and pO_2_ levels, but unfortunately these studies were mainly performed in the non-compromised foetus [25–27]. Furthermore, only a few non-randomised studies of poor quality have been performed in the distressed foetus [28–32]. These studies suggest an improvement in FHR patterns and foetal scalp pH when 100% oxygen is applied to the mother. Based on these publications, authors of a Cochrane review published in 2012 concluded that ‘there is not enough evidence to support the use of prophylactic oxygen therapy for women in labour, nor to evaluate its effectiveness for fetal distress’, owing to the lack of randomised controlled trials (RCTs) [33].

An important concern in the use of maternal hyperoxygenation for foetal distress is the potential negative effect on umbilical cord pH. In a study by Thorp et al. [34], 86 term parturients were randomised to receive additional oxygen or normal care during the second stage of labour. The main outcome measures were cord blood gas and co-oximetry values. The mean cord blood gas values did not significantly differ between the intervention and control groups. However, Thorp et al. found significantly more arterial pH values < 7.20 in the group receiving extra oxygen. The lowest pH in arterial blood gas (pHa) value that they found was 7.09. They also found that the duration of oxygen therapy was inversely related to arterial cord pH, whereas Apgar scores and hospital admission rates did not differ between the groups. They concluded that prolonged oxygen treatment during the second stage of labour leads to a deterioration of cord blood gas values at birth. An important fact is that only patients with reassuring FHR patterns were included in their study. Therefore, (ominous) foetal hypoxia at the start of oxygen delivery was very unlikely. Thus, their study did not address the effect of maternal hyperoxygenation in cases of suspected foetal distress.

Another frequently stated argument to discourage maternal hyperoxygenation as standard care is the potential increase in free oxygen radicals in both mother and foetus [35, 36]. An increase in the markers for free oxygen radical production has been seen for the use of high fractions of inspired oxygen and in the presence of non-reassuring FHR patterns [35–38]. Also, lipid peroxide concentrations in arterial cord blood are higher after uncomplicated vaginal delivery than after elective caesarean section [39].

To a certain degree, free oxygen radicals are physiological and known to be higher in the presence of several maternal and foetal conditions, such as pre-eclampsia, diabetes, smoking, intrauterine growth restriction and foetal distress [37, 39–42]. The effect of maternal hyperoxygenation on free oxygen radical release, in response to non-reassuring foetal status, has not yet been investigated.

What we do know is that neonatal resuscitation with 100% oxygen may lead to an increase in neonatal mortality and morbidity, including bronchopulmonary disease and retinopathy, mainly in premature infants [43–46]. However, the increase in foetal pO_2_ due to maternal hyperoxygenation will never reach the levels obtained by the direct application of 100% oxygen directly to the foetus [23]. To our knowledge, the clinical implication of increased free radical production due to maternal hyperoxygenation has not been investigated. Researchers in studies using maternal hyperoxygenation as a treatment for the growth restricted foetus did not report any harmful effects [47, 48].

With regard to the mother, some potential side effects have to be taken into account. The use of high fractions of inspired oxygen in the absence of tissue hypoxia may cause toxic effects as a result of oxidative stress [49, 50]. This may lead to, for example, mucosal inflammation, hypoperfusion and pneumonitis [51]. A reversible vasoconstriction of approximately 10% in the maternal brain has been described [52]. However, this is not expected to cause any harm [53, 54]. Administration of 100% oxygen during labour is not investigated. However, it has been well investigated for the treatment of cluster headaches, and no severe side effects (e.g., hypoventilation and fainting) have been reported [54].

Inhaling high fractions of inspired oxygen will increase the concentration of free oxygen radicals in maternal blood [35]. Despite the adverse effects of free oxygen radicals that have been described [55], it is unlikely these will cause clinically relevant tissue damage, owing to the mature anti-oxidant system in the adult [35, 36]. Also, the Dutch Pharmacovigilance Centre Lareb has not been informed of any side effects of oxygen therapy [56].

### Current recommendations on the use of maternal hyperoxygenation

On the basis of current knowledge, it is difficult to determine whether the beneficial effects of maternal hyperoxygenation outweigh the potential side effects. As a consequence, recommendations in international guidelines and use in clinical practice are non-uniform [20]. Maternal hyperoxygenation during labour is often used in the United States to increase oxygen transport towards the foetus [21]. The American College of Obstetricians and Gynecologists guideline on foetal resuscitation recommends the administration of oxygen to the mother in cases of foetal distress [57]. In contrast, the Royal College of Obstetricians and Gynaecologists explicitly states in their Green Top Guideline not to apply maternal oxygenation for reasons other than maternal hypoxia, until the beneficial effect is proven [58]. A recent discussion on benefit and harm of maternal hyperoxygenation in the *American Journal of Obstetrics and Gynecology* emphasised the current lack of evidence [21–23]. In fact, several reviews underline an urgent need for an RCT investigating the effect of maternal hyperoxygenation on the foetal condition [21–23, 33].

## Methods/design

### Aim

The aim of this study is to investigate the effect of maternal hyperoxygenation with 100% oxygen on the foetal condition during the second stage of labour in the presence of suspected foetal distress during term labour. Also, we will investigate the potential side effects to formulate recommendations for international clinical practice and future research.

### Study design

**Fig. 1:**
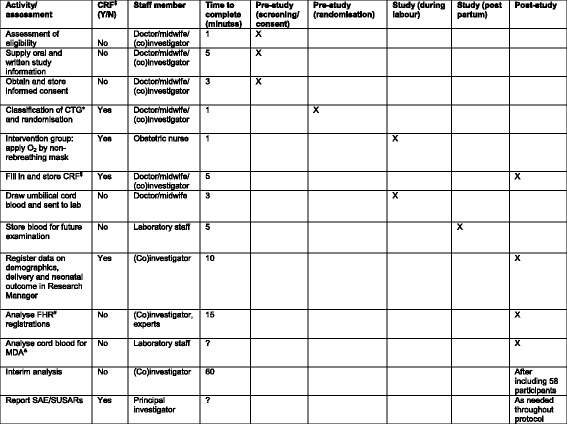
The schedule of forms and procedures, according to the Standard Protocol Items: Recommendations for Interventional Trials (SPIRIT). *CRF* Case report form, *CTG* Cardiotocogram, *FHR* Foetal heart rate, *MDA* Malondialdehyde, *SAE* Serious adverse event, SUSAR Suspected unexpected serious adverse reaction

This study will be a single-centre RCT performed in a tertiary hospital in The Netherlands. We are comparing maternal hyperoxygenation for the treatment of foetal distress during the second stage of labour with conventional care. All procedures and time frames are displayed in Fig. 1 according to the Standard Protocol Items: Recommendations for Interventional Trials (SPIRIT) [59]. Additional file 1 contains the complete SPIRIT checklist.

### Participants

The study population will be drawn from among parturients admitted to the labour ward of a tertiary hospital (Máxima Medical Centre, Veldhoven, The Netherlands), where approximately 2200 deliveries occur annually, of which approximately 1900 are term births. CTG and, if necessary, FSBS are generally used for foetal monitoring during labour. Maternal repositioning, discontinuation of administration of oxytocin, use of tocolytic drugs and intermittent pushing are common interventions used to achieve intrauterine resuscitation, whereas amnioinfusion and maternal hyperoxygenation are never applied as standard care in our centre.

### Inclusion criteria

Pregnant women ≥ 18 years old in term labour and with an intended vaginal delivery of a singleton in cephalic presentation can participate in this study.

### Exclusion criteria

Exclusion criteria are determined with a focus on the risk of excessive production of free oxygen radicals and reducing the influence of other factors affecting FHR pattern. These are recent use of any of the following medications: corticosteroids, anti-hypertensives, magnesium sulphate, amiodarone, opioids, adriamycin, bleomycin, actinomycin, menadione, promazine, thioridazine or chloroquine. Other exclusion criteria are the use of tobacco, recreational drugs or alcohol during pregnancy. Parturients with pre-existing cardiac disease, pulmonary disease with the use of medication, diabetes, hyperthyroidism or anaemia (haemoglobin < 6.5 mmol/L or 10.5 g/dl) will also be excluded. Foetal factors leading to exclusion are suspected infection during labour (need for antibiotics), congenital malformations and normal or pre-terminal FHR pattern, or prolonged bradycardia (according to the modified International Federation of Gynecology and Obstetrics [FIGO] classification; *see* Table 1) [60, 61].

**Table 1:** Classification of cardiotocograms according to the modified International Federation of Gynecology and Obstetrics criteria

	Baseline heart frequency	Variability Reactivity	Decelerations
Normal CTG	• 110–150 bpm	• Accelerations • 5–25 bpm	• Early uniform decelerations • Uncomplicated variable decelerations (loss of < 60 beats)
Intermediary CTG	• 100–110 bpm • 150–170 bpm • Short bradycardia episode < 100 bpm for > 3 minutes < 80 bpm for > 2 minutes	• > 25 bpm (saltatory pattern) • < 5 bpm > 40 minutes	• Uncomplicated variable decelerations (loss of > 60 beats)
	• A combination of two or several intermediary observations will result in an abnormal CTG		
Abnormal CTG	• > 170 bpm • Persistent bradycardia < 100 bpm for > 10 minutes < 80 bpm for > 3 minutes (without an increasing tendency)	• < 5 bpm for > 60 minutes • Sinusoidal pattern	• Complicated variable decelerations with a duration of > 60 seconds • Repeated late uniform decelerations
Pre-terminal CTG	• Total lack of variability (< 2 bpm) and reactivity with or without decelerations or bradycardia		

*bpm* Beats per minute, *CTG* Cardiotocogram

### Patient recruitment and randomisation

All patients eligible to be included in this study will be asked antepartum to participate when they visit the outpatient clinic or when they are admitted to the delivery ward. All patients will receive oral and written information about the study from the attending midwife or doctor or from a co-investigator (Additional files 2 and 3). After informed consent is obtained, and only in case of suboptimal or abnormal FHR patterns during the second stage of labour, randomisation is performed using sealed opaque envelops. The allocation sequence is computer-generated using random blocks of four or six patients.

### Intervention and control groups

Patients will randomly be assigned to one of the two arms of the study:

1. *Control group*: Normal care (according to local standards) is provided, and preferably started at least 10 minutes after the onset of a suboptimal or abnormal FHR pattern, according to the modified FIGO criteria (Table 1) [60, 61].
2. *Intervention group*: In case of a suboptimal or abnormal FHR pattern according to the modified FIGO criteria, 100% oxygen is applied to the mother at 10 L/minute via a non-rebreathing mask and continued until delivery. Co-interventions (normal care) may be initiated after 10 minutes of oxygen administration without a satisfactory effect on FHR to investigate the effect of only maternal hyperoxygenation on FHR, without risking prolonged foetal hypoxia. In case a patient needs to undergo a caesarean section, oxygen administration will be continued until the baby is born.

Obviously, in any case where the delivery room staff believe additional interventions should be applied for safety reasons, the study protocol can be overruled at any time.

### Study outcomes and data analysis

The primary outcome is the percentage reduction in the depth and duration of FHR deceleration in the intervention group compared with the control group. Secondary outcomes include foetal, neonatal and maternal outcomes.

#### Foetal outcome

1. *FHR changes*: Changes in specific features of the CTG, including the following:
  - Decelerations with loss of internal variability (beat-to-beat variability of < 5 beats per minute [bpm])
  - Decelerations in combination with tachycardia of bradycardia (> 160 or < 110 bpm)
  - Unassignable baseline
  - Phase-rectified signal averaging (PRSA), a relatively new technique used to determine FHR variability by estimating the accelerative capacity (AC_PRSA_) and decelerative capacity (DC_PRSA_) of the foetal heart. This technique is explained in articles by Bauer and Huhn [62, 63].
2. *Change in modified FIGO classification* (Table 1) [60, 61].

In the next subsection, methodology regarding the comparison of FHR tracings and timeframes is described in more detail.

#### Neonatal outcome

Neonatal outcome includes Apgar score, NICU admission, venous and arterial umbilical cord blood gas analysis (pH, lactate, base excess, pO_2_ and pCO_2_) and malondialdehyde (MDA, a marker for free oxygen radical production) in arterial and venous umbilical cord blood. Information on neonatal admission is a standard part of the maternal hospital chart. Determination of 1- and 5-minute Apgar scores and venous and arterial umbilical cord blood gas analysis (pH, lactate, base excess, pO_2_ and pCO_2_) is common practice. Cord blood gas analysis will be performed immediately after birth using the ABL 90 flex blood gas analyser (Radiometer Benelux BV, Zoetermeer, The Netherlands) with both venous and arterial cord blood. Two additional blood samples (one venous and one arterial sample) are drawn from the umbilical cord in heparinised tubes and immediately centrifuged and stored at the laboratory of Máxima Medical Centre at − 20 °C. Once all samples are collected, they will be transported to the Laboratory of Genetic and Metabolic Diseases of the Academic Medical Centre Amsterdam (Amsterdam, The Netherlands), where total (free and bound) MDA will be determined as the 2,4-dinitrophenylhydrazine (DNPH) derivative. A stable isotopically labelled analogue (^2^H_2_-MDA) will be added as an internal standard, then alkaline hydrolysation, deproteinisation and derivatisation with DNPH, and MDA-hydrazone will be analysed by high-performance liquid chromatography-tandem mass spectrometry and positive electrospray. Samples will be injected on a SUPELCOSIL LC-18-DB analytical column (250 × 4.6 mm, 5-μm particles; Sigma-Aldrich, St. Louis, MO, USA) and will be separated using an ACQUITY ultra performance liquid chromatography system (Waters, Milford, MA, USA). Samples will then be analyzed by a Quattro Premier XE mass spectrometer (Waters, Milford, MA, USA). Analytes and internal standards will be eluted with acetonitrile/water/acetic acid (50/50/0.2) and detected in multiple reaction monitoring mode for the transitions of mass-to-charge ratio (*m/z*) 235 → *m/z* 159; *m/z* 237 → *m/z* 161.

#### Maternal outcome

Maternal outcome measures include the mode of delivery, side effects and reasons for discontinuation of oxygen administration. Side effects include headache, dizziness, discomfort of the non-rebreathing mask and any other complaint mentioned by the participant. The delivery room staff will register on the case report form if the parturient experiences any side effects and/or if there are reasons for eventual discontinuation of oxygen administration. Also, to gain insight into how labouring women experience receiving additional oxygen via a non-rebreathing mask compared with receiving normal care, a short questionnaire will be used to investigate experiences of all the participants with this study.

### Analysis of outcome measures regarding FHR pattern

#### Changes in FHR pattern

The digital CTG tracings will be extracted from CS-EZIS (ChipSoft, Amsterdam, The Netherlands) and analysed using MATLAB 2015a software (MathWorks Inc., Natick, MA, USA). For the computerised CTG analysis, we will use a custom-made algorithm based on the OxSys system [64] that will first be validated by an expert panel. This expert panel will also manually classify the CTG into one of the FIGO categories [60, 61]. Regarding the analysis of specific CTG features, we searched the literature for CTG features that are likely related to neonatal outcome. A large variety of CTG features have been investigated in relation to neonatal outcome, with varying results. However, three features are consistently mentioned as being related to neonatal outcome: decelerations with loss of internal variability, decelerations in combination with tachycardia or bradycardia and periods with unassignable baseline [3, 60, 64–71]. Besides, AC_PRSA_ and DC_PRSA_ turned out to predict acidaemia better than short-term variation [62, 72, 73]. We therefore include this parameter as an outcome measure.

#### What is the time frame of interest?

All patients serve as their own control, with changes in FHR being compared before and after the start of the study protocol, regardless of whether the patients belong to the control or the intervention group. Results in the intervention group and the control group will also be compared.

**Fig. 2:**
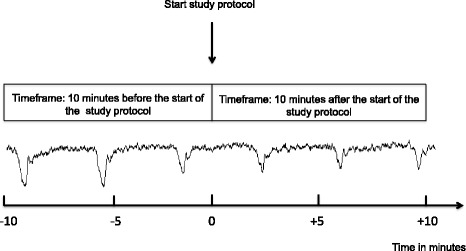
The time frame of interest for analysis of outcome measures where patients serve as their own control: the control group. bpm, Beats per minute; CTG, Cardiotocogram

**Fig. 3:**
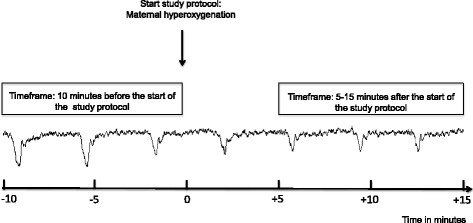
The time frame of interest for analysis of outcome measures where patients serve as their own control: the intervention group

For the analysis where patients serve as their own control, the time frames of interest for outcomes related to changes in FHR are as follows:

1. *Control group*: 10 minutes before and after the start of the study protocol. In total, 20 minutes of data will be analysed (Fig. 2).
2. *Intervention group*: 10 minutes before the start of the study protocol up to 15 minutes after the start of the study protocol. The time frame of interest after the start of the study protocol is determined as the period between 5 and 15 minutes after maternal hyperoxygenation is initiated, motivated by the expectation that it will take 5 minutes for maternal pO_2_ to increase to a maximum of approximately 475 mmHg [24]. After that, the effect of the intervention will be observed for 10 minutes. In total, 20 minutes of data will be analysed (Fig. 3).

These periods are established because during this period, maternal hyperoxygenation can be compared with no treatment. Furthermore, we will also compare the periods from the start of the study until birth, although these results may be influenced by other interventions that may have been applied.

#### Other study endpoints and parameters

Other study endpoints and parameters to be recorded are duration of the second stage of labour, duration of time for which supplemental oxygen was received, baseline characteristics (infant sex, gestational age and birth weight, maternal age and parity).

### Hypothesis

We hypothesise that maternal hyperoxygenation will improve FHR, without any severe maternal side effects. We do not expect a difference in rates of vacuum-assisted delivery or secondary caesarean sections or in Apgar scores or umbilical cord pH values, owing to the relatively small sample size. Furthermore, we expect larger concentrations of MDA in the intervention group than in the control group.

### Handling and storage of data and documents

Data will be handled anonymously, and we will adhere to the Dutch Personal Data Protection Act. A secured subject identification code list will be used to link a study number to a patient’s name and date of birth. This file is password-protected and available only to the main investigator (LMB). All other information will contain only the study number and no data directly referring to the patient. Foetal blood gas values will be stored in the neonates’ hospital charts because this is part of conventional care. Laboratory results regarding markers for free oxygen radicals will be coded and will therefore be anonymous. All data will be stored for 15 years in accordance with good clinical practice guidelines.

### Statistical analysis

#### Sample size calculation

The study consists of two study groups: one group with suboptimal FHR patterns and one group with abnormal FHR patterns. We aim for the study to have 80% power and a level of significance of 0.05 in both groups. In one small, non-randomised study, a reduction in FHR decelerations (type II dips) of 50–100% was noted. Our present study is the only one to report FHR changes as a result of maternal hyperoxygenation. On the basis of available literature, we expect ≥ 50% improvement in the oxygen group and 0% in the control group in both suboptimal and abnormal FHR patterns [28]. We estimated a mean improvement of 50% with an SD of 50% in each group. A power analysis performed in G*Power 3.0.10 (Kiel University, Kiel, Germany) for a two-tailed Mann-Whitney *U* test (assuming that data will not be equally distributed) resulted in a sample size of 67 patients in each study group. We added an extra 20% to compensate for missing data. Because we have two separate study groups (suboptimal and abnormal FHR group), we need 162 patients to participate.

#### Data analysis

IBM SPSS Statistics software (version 24; IBM, Armonk, NY, USA) will be used to perform statistical analysis of the study results. Assuming non-normal distribution, the primary clinical outcome will be analysed with a Mann-Whitney *U* test for differences between the intervention and control groups and a Wilcoxon matched-pairs test for changes within the same participant. When outcome data are found to be normally distributed, independent samples *t* tests (two-tailed) will be used to analyse differences between the intervention and control groups, and paired *t* tests will be used for changes within the same participant. Outcome measures will be calculated for the combined group and the subgroups of suboptimal and abnormal FHR tracings, as well as for small for gestational age (SGA, lower than 10th percentile) and appropriate for gestational age (AGA) neonates. In the intervention group, oxygen may not be applied, owing to practical concerns such as very quick progression of labour. Therefore, we will perform both per-protocol and intention-to-treat analyses. In the per-protocol analysis, parturients who actually received oxygen will be compared with those who did not receive oxygen. In addition, unjust inclusions will be excluded from this analysis.

#### Interim analysis

On account of safety concerns, an interim analysis will be performed when 50% of the patients are included in the study. In this analysis, we will compare the number of neonates with a 5- minute Apgar score < 7 and/or pHa < 7.05, the number of admissions to the NICU and perinatal death in both groups (all neonates that received oxygen in both suboptimal and abnormal CTG groups versus ‘conventional care’ group). If the interim analysis shows a significant difference, we will terminate the study. This interim analysis is performed exclusively for safety reasons; because the primary outcome measure (FHR) will not be analysed during the interim analysis and power analysis is based on the primary outcome, adjustment of the significance level is not required.

### Public disclosure and publication policy

All investigators agree to publish the study results in an international peer-reviewed journal, even if the results do not correspond to the hypothesis as stated in the Methods section of the protocol. The results will be offered for publication after all the investigators agree on the content of the article. The full protocol (version 8, dated 1 March 2017) is available upon request.

## Discussion

This study is the first RCT to investigate the effect of maternal hyperoxygenation for foetal distress during labour [18, 33]. So far, the effects of supplemental oxygenation in the presence of FHR abnormalities have been investigated only in small, non-randomised studies. Because of the lack of concrete results from clinical trials, it is hard to compare the beneficial effects of maternal hyperoxygenation with the potential side effects. As a result, recommendations on the use of this intervention for foetal distress in international guidelines are non-uniform [20]. Thus, the results of this study will help to filling an internationally recognised ‘research gap’.

We believe patient safety is carefully addressed in this study, and ethical concerns are limited. One of the major concerns of administering high fractions of oxygen is the increase in free oxygen radicals. Whether this has a clinical effect remains unclear. We excluded from this study all patients with a higher a priori risk of exposure to increased free oxygen radical levels.

Both practical and safety issues led to limitations of this study. An important limitation is the primary outcome measure. We recognise that changes in FHR as a primary outcome measure are not optimal, because FHR does not accurately reflect foetal oxygenation and acid-base balance [60, 74, 75]. However, we believe this is the best available method to record changes in the foetal condition during labour. Furthermore, we assume that if no beneficial effect on FHR can be shown, an improvement in neonatal outcome is unlikely. Ideally, neonatal outcome measures such as Apgar score and umbilical cord pH are the outcome measures of first choice. However, a study with appropriate power to address these outcome measures would need a very large sample size. Because the potentially harmful effects have not been investigated properly yet, we chose not to expose a large group of women and their foetuses to this intervention. If a positive effect on FHR pattern without severe side effects can be confirmed by this study, we will perform a larger multicentre RCT to investigate the effect on Apgar score and cord blood gas values.

In this study, we focus on the foetal condition during the second stage of labour and short-term neonatal outcome. This implies that abnormalities in FHR patterns during the first stage of labour are not taken into account. We believe that the randomisation process will limit its influence. With regard to the neonatal period, we did not arrange long-term follow-up, because we do not expect any clinically relevant side effects that can be attributed to maternal hyperoxygenation. Besides, the sample size is too small to draw firm conclusions on long-term neonatal effects in this study.

Power analysis of the present study is based on the expected effect on the primary outcome measure, and the study is not powered to find any significant differences in Apgar score and umbilical cord blood gas values. In the power analysis, we used an expected improvement in deceleration depth and duration of 50%. On one hand, this value is based on small non-randomised studies and may be overestimated. On the other hand, those studies provide the only available data. Also, we believe it is unlikely that a limited improvement in deceleration depth and duration has clinical relevance. The sample size is calculated for each of the subgroups of suboptimal and abnormal FHR tracings. We believe it is important to assess the effect of the intervention in these subgroups because foetuses with lower initial pO_2_ levels may profit more from maternal hyperoxygenation [29].

Regarding the subgroups of AGA and SGA infants, we did not increase our sample size to reach an adequate number of participants in the SGA group. Nevertheless, we find it interesting to investigate whether there is a different effect of maternal hyperoxygenation in SGA infants compared with AGA infants.

Because of organisational challenges, it is not possible to conduct a double-blind trial. Hence patients and delivery room staff are not blinded to the patient’s allocation to a study group, which may lead to observer bias. However, analysis of FHR tracings will be done using a computerised algorithm, to minimise bias, and the investigators judging the CTGs and secondary outcome measures are blinded to the study arm.

To investigate the effect of maternal hyperoxygenation in the presence of foetal distress on the release of free oxygen radicals, MDA is estimated in umbilical cord blood. MDA is the peroxidation product of membrane polyunsaturated fatty acids. We chose to measure this marker for oxidative stress because it was used in prior studies performed during labour and it is related to vaginal birth, non-reassuring FHR tracings, maternal hyperoxygenation and acidaemia in arterial cord blood [36, 37, 39, 41]. We realise that differences in values in umbilical cord blood may be confounded by mode and duration of delivery; therefore, we will correct the results for the mode of delivery. A practical ground on which to choose this marker is that it is the only marker for oxidative stress that can be estimated accurately in Dutch laboratories. In the intervention group, oxygen administration will be continued until delivery to enable analysis of its effect on cord blood gas values and MDA.

Despite some important limitations of this study, we believe this is the best possible way to perform a study while restricting safety issues. If the results do not show any improvement in FHR, we believe that maternal hyperoxygenation should not be used as a treatment for foetal distress. However, if a beneficial effect is demonstrated, we will design a multicentre RCT to investigate the effect on neonatal outcome.

## Trial status

Patient recruitment started on 1 March 2016 and was estimated to be fulfilled on 31 March 2018.

## Additional files

Additional file 1: SPIRIT 2013 checklist: recommended items to address in a clinical trial protocol and related documents. (PDF 23602 kb)

Additional file 2: Information and informed consent in English. (DOCX 23 kb)

Additional file 3: Information and informed consent in Dutch. (DOCX 28 kb)

## Abbreviations

AGA: Appropriate for gestational age
bpm: Beats per minute
CTG: Cardiotocogram
DC_PRSA_: Phase-rectified signal-averaging decelerative capacity
DNPH: 2,4-Dinitrophenylhydrazine
FHR: Foetal heart rate
FIGO: International Federation of Gynecology and Obstetrics
FSBS: Foetal scalp blood sampling
MDA: Malondialdehyde
*m/z*: Mass-to-charge ratio
NICU: Neonatal intensive care unit
pCO_2_: Partial carbon dioxide pressure
pHa: pH in arterial blood gas
pO_2_: Partial oxygen pressure
PRSA: Phase-rectified signal averaging
RCT: Randomised controlled trial
SAE: Serious adverse event
SGA: Small for gestational age
SPIRIT: Standard Protocol Items: Recommendations for Interventional Trials
SpO_2_: Oxygen saturation
SUSAR: Suspected unexpected serious adverse reaction
