# Correction to: Intrauterine resuscitation during the second stage of term labour by maternal hyperoxygenation versus conventional care: study protocol for a randomised controlled trial (INTEREST O2)

**DOI:** 10.1186/s13063-018-2963-2

**Published:** 2018-10-23

**Authors:** Lauren M. Bullens, Alexandra D. J. Hulsenboom, Suzanne Moors, Rohan Joshi, Pieter J. van Runnard Heimel, M. Beatrijs van der Hout-van der Jagt, Edwin R. van den Heuvel, S. Guid Oei

**Affiliations:** 1 Department of Obstetrics and Gynaecology, Máxima Medical Centre, PO Box 7777, 5500 MB Veldhoven, The Netherlands; 2 Department of Electrical Engineering, Eindhoven University of Technology, PO Box 513, 5500 MB Eindhoven, The Netherlands; 3 Department of Clinical Physics, Máxima Medical Centre, PO Box 7777, 5500 MB Veldhoven, The Netherlands; 4 Department of Industrial Design, Eindhoven University of Technology, PO Box 513, 5500 MB Eindhoven, The Netherlands; 5 Department of Mathematics and Computer Science, Eindhoven University of Technology, PO Box 513, 5500 MB Eindhoven, The Netherlands

## Correction

Following publication of the original article [1], the authors noticed that the sample size for the study group was incorrectly reported in the Methods section. The correct number of patients to participate in the study is 116.
