# Supplementary material for: Intrauterine resuscitation during the second stage of term labour by maternal hyperoxygenation versus conventional care: study protocol for a randomised controlled trial (INTEREST O2)
Source: Trials. 2018 Mar 23;19:195. doi: 10.1186/s13063-018-2567-x (PMC5865381; doi:10.1186/s13063-018-2567-x)
Supplement: Supplementary file 2 — Information and informed consent in English. (DOCX 23 kb) [file 13063_2018_2567_MOESM2_ESM.docx]

**Information for participants**

*Can fetal distress during labor be treated by administering additional oxygen to the mother?*

*(INTEREST-O2)*

Dear Ms,

You are, or will be, administered at Máxima Medical Centre because you are going to give birth to your baby here. During the delivery we will continuously monitor the heartbeat of your baby to estimate his/her wellbeing.

Of course we expect that the delivery will go well. Sometimes, however, the baby’s heartbeat changes; this can be an indication that the baby is not receiving enough oxygen. This usually happens at the final stage of childbirth when the effect of the contractions on the baby is the greatest. This last stage of birth is called the pushing stage.

If this is the case, it is necessary to start treatment to prevent prolonged periods of oxygen deficiency. Oxygen deficiency may have negative effects on the baby’s development after birth.

When there is a suspicion of oxygen deficiency, we will try to increase the oxygen flow to the baby, to restore oxygen levels. Various treatments can be initiated to treat oxygen deficiency. When the indications of oxygen deficiency are severe, we sometimes choose to deliver the baby immediately, through a caesarean section or vacuum assisted vaginal delivery.

Examples of standard treatments to restore the baby’s oxygen levels are: turning you on your other side, discontinuation of contraction stimulating medication (if these are administered), stop pushing or administering medication to stop contractions.

In the United States and a lot of Dutch hospitals, there is another option for standard treatment, namely administering extra oxygen to the mother. The additional oxygen that the mother inhales through a cap on her nose and mouth will go to the baby, via the placenta. The baby’s, heartbeat can recover and therefore probably the baby’s condition. In the Netherlands this is not a standard treatment yet.

**What is the purpose of this study?**

With this study we want to investigate if the administration of additional oxygen to the mother will reduce oxygen distress for the baby during the last stage of labor. We want to examine a number of aspects with this research: Firstly we want to see if the heartbeat of the baby returns back to normal more quickly if oxygen is administered. In addition, we will determine values that reflex oxygen status in the umbilical cord blood shortly after birth.. We also want to determine an extra value in the blood of the umbilical cord that will show us how much ‘stress’ the baby endured during labour. Finally, we would like investigate how women experience participating in this study.

**What does participation in the study mean?**

You can be included in the programme if the heartbeat of the baby changes during the pushing stage and might indicate oxygen shortage. If you are getting the treatment with extra oxygen, or if you are in the control-group is randomly decided. If you are in the control-group this means you will receive a standard treatment mentioned earlier. If you receive the treatment with extra oxygen, this will be administered through a cap enclosing your nose and mouth. When breathing through the cap you will inhale 100% oxygen. The nurse will help you with this. The treatment continues until the baby is born. If the heartbeat pattern is not reassuring, you will receive the standard treatment as well. There is no risk that oxygen deficiency of the baby is left untreated. Your doctor or midwife will decide which treatments are necessary to ensure the optimal condition of your baby. If, despite all treatments, the heartbeat is not reasurring, the doctor will make sure the baby is delivered quickly by means of a caesarean section or vacuum assisted vaginal delivery, like normal.

After the delivery blood will be collected from the umbilical cord. In this blood we will determine a number of values that reflect the oxygen levels the baby had prior to birth. This is part of the standard treatment and both you and the baby will not notice any of this. For this research we want to collect additional blood from the umbilical cord to determine other levels in the blood that indicate how much ‘stress’ the baby has experienced during childbirth. This procedure is also not noticeable for you or the baby.

Naturally you will get time to consider whether you want to participate in this programme. For additional information, you can take a look at the brochure "Medical research." You can decide up until with the start of the last stage of childbirth. It won’t cost you any extra time and there will be no extra appointments in the hospital. However, we do ask you to fill in a short multiple-choice questionnaire on how you experienced participating in this study. You can fill it in in the hospital or at home.

**What are the benefits in participating in this research programme?**

Previous studies show that is it likely that additional oxygen given to the mother increases the oxygen levels in the baby and that the heartbeat pattern improves. We want to confirm this with a study of better quality than that studies that have been done already by adding a control-group. It could be that if the heartbeat recovers, the doctor will not have to use invasive treatment such as a caesarean section or vacuum assisted vaginal delivery. Not using these invasive treatments is beneficial for you and the baby, because there are health risks associated with both.

Besides the advantages participating in this study for you, it is also important for other pregnant women in the future. The results of the study will be published. If oxygen administration is proven to improve the condition of the baby during the delivery, other women and their babies can profit from it as well. This allows improvements in birth care in The Netherlands and worldwide.

**Are there any risks?**

We do not expect any large risks for you or your baby when you participate in this research programme. 100% oxygen is administered regularly in other healthy adults for different medical purposes (for example treatment of severe headaches), and there are no serious side effects known. It is known, however, that when the mother receives additional oxygen, the level of specific particles, the reactive oxygen species, increases. These particles are necessary for ‘normal’ processes in the human body, but can do harm if they exist in large quantities. The number of particles also increases if there is an oxygen deficiency, even if the mother does not receive additional oxygen. We do not expect that the build-up of these particles will do any damage to your baby. In the United States millions of mothers receive additional oxygen during childbirth every year without causing problems to the newborn baby.

**What will be different in contrast to not participating?**

If you will participate with this research there will be two things different than when you get the ‘standard’ treatment. If the computer program decides that you are in the group that will receive additional oxygen, this deviates from the standard treatment in the Netherlands. It can also be that you will be part of the control-group, this group receives the same treatment as non-participants. Regardless of in which group you are placed, two extra tubes of blood will be collected from the umbilical cord.

**What will happen if you decide not to participate in this research programme?**

If you do not wish to participate you will receive standard medical treatment during childbirth. Participation is entirely voluntary. At any time you can withdraw from this programme, even if you already pledged participation. This does not have any consequences for further treatments. You are not obliged to give the reason for withdrawal.

**Are you insured when you participate in the research programme?**

There is insurance for patients participating in this research at MediRisk.

**What happens to your personal data?**

Your personal data will be handled confidentially. The data that is collected especially for part of the study are only insightful for the researchers named below. We have to obey the law for protection of personal information. It is mandatory to save your data for 15 years. If you participate with this research, you will automatically give permission to do so.

**Will your general practitioner and/or medical specialist be informed if you participate in the research programme?**

No, your general practitioner and/or medical specialist will not be informed because we do not expect the treatment will give any disadvantages to your health. The gynaecologists, gynaecologists in training and midwives in the Máxima Medical Centre will know that you are participating.

**Are there any extra cost/compensation if you participate in the research programme?**

No, you won’t receive compensation by participating in the research. This research is completely voluntary.

**Who approves this research programme?**

The central Committee Human Research and the Dutch Ministry of Health, Welfare and Sports have approved the execution of this research programme. In the brochure “Medical research”, you can find more information about this.

**More questions?**

If you have more questions after reading this information, please contact one of the researchers listed below. Besides the researchers there is an independent expert who is available for answering your questions concerning the research. This independent person is a doctor who is not involved in the research, but is knowledgeable on the subject. His contact details are listed below.

**What can you do if you have a complaint?**

If you are unhappy with the research, there is an independent complaints committee of Máxima Medical Centre. You can reach the committee on the cell phone number: 040-8889115.

**Researchers involved in this project**

Lauren Bullens, M.D., Máxima Medical Centre

Olenka Hulsenboom, M.D., Máxima Medical Centre

Suzanne Moors, M.D., Zuyderland Medical Centre

Bernice Wieland, BSc, Máxima Medical Centre

Beatrijs van der Hout-ven der Jagt, MSc, PhD, Máxima Medical Centre

Pieter van Runnard Heimel, M.D., PhD, gynaecologist-perinatologist, Máxima Medical Centre

Prof. Guid Oei, M.D., PhD, gynaecologist-perinatologist, Máxima Medical Centre

Phone: 040-8889551 (delivery rooms)

Email: lauren.bullens@mmc.nl

**Independent researcher**

Peter Andriessen, M.D., PhD, pediatrician-neonatologist

Email: [p.andriessen@mmc.nl](mailto:p.andriessen@mmc.nl)

Phone: 040-8889350

**Attachments**

- Written declaration of consent
- Brochure “Medical research”

NL53018.000.15 Informed consent form (copy for participant)

**Written declaration for consent**

*Can fetal distress during labor be treated with additional oxygen administration to the mother?*

*(INTEREST-O2)*

I have read the information letter for participants. I had the chance to ask additional questions. My questions have been adequately answered. I had enough time to decide if I would participate. I know that participating is entirely voluntary. I know that I can decide to withdraw from the study at any moment. I do not have to give my reason for withdrawal.

I know researchers can look in my data. I give permission to use the data for the intentions written in the information letter. I give permission to save my research data 15 years after the end of the study.

I want to participate in this research.

Name participant:

Signature: Date: ___ / ___ / ___

**------------------------------------------------------------------------------------------------------**

I declare that I fully informed the participant about the research mentioned. If something changes during the study that can affect the consent of the participant, I will notify the participant timely.

Name researcher (or his representative):

Signature: Date: ___ / ___ / ___

--------------------------------------------------------------------------------------------------------

Additional information is given by (if applicable):

Name:

Function:

Signature: Date: ___ / ___ / ___

NL53018.000.15 Informed consent form (copy for research file)

**Written declaration for consent**

*Can fetal distress during labor be treated with additional oxygen administration to the mother?*

*(INTEREST-O2)*

I have read the information letter for participants. I had the chance to ask additional questions. My questions have been adequately answered. I had enough time to decide if I would participate. I know that participating is entirely voluntary. I know that I can decide to withdraw from the study at any moment. I do not have to give my reason for withdrawal.

I know researchers can look in my data. I give permission to use the data for the intentions written in the information letter. I give permission to save my research data 15 years after the end of the study.

I want to participate in this research.

Name participant:

Signature: Date: ___ / ___ / ___

**------------------------------------------------------------------------------------------------------**

I declare that I fully informed the participant about the research mentioned. If something changes during the study that can affect the consent of the participant, I will notify the participant timely.

Name researcher (or his representative):

Signature: Date: ___ / ___ / ___

--------------------------------------------------------------------------------------------------------

Additional information is given by (if applicable):

Name:

Function:

Signature: Date: ___ / ___ / ___
