# Supplementary material for: Intrauterine resuscitation during the second stage of term labour by maternal hyperoxygenation versus conventional care: study protocol for a randomised controlled trial (INTEREST O2)
Source: Trials. 2018 Mar 23;19:195. doi: 10.1186/s13063-018-2567-x (PMC5865381; doi:10.1186/s13063-018-2567-x)
Supplement: Supplementary file 3 — Information and informed consent in Dutch. (DOCX 28 kb) [file 13063_2018_2567_MOESM3_ESM.docx]

**Informatie voor proefpersonen**

*Kan zuurstofnood van het ongeboren kind tijdens de bevalling worden behandeld door het toedienen van extra zuurstof aan de moeder?
(INTEREST-O2)*

Geachte mevrouw,

U bent of wordt binnenkort in het ziekenhuis opgenomen omdat u gaat bevallen in Màxima Medisch Centrum. Tijdens uw bevalling zullen wij voortdurend het hartslagpatroon van uw baby in de gaten houden om te controleren of de baby de weeën goed verdraagt.

Wij verwachten uiteraard dat de bevalling goed zal verlopen, maar soms merken we aan het hartslagpatroon van de baby dat er mogelijk sprake is van zuurstofgebrek. Dit gebeurt meestal tijdens de het laatste stukje van de bevalling, omdat tijdens het persen de belasting voor de baby het grootst is. Dit laatste gedeelte van de bevalling heet de uitdrijving.

Het is nodig om dan een behandeling te starten, om te voorkomen dat er langdurig sprake is van zuurstofgebrek, wat negatieve gevolgen kan hebben voor de ontwikkeling van de baby na de geboorte.

Er zijn verschillende behandelingen die we kunnen starten. Als er een verdenking bestaat op zuurstofgebrek, proberen we om door een bepaalde behandeling de zuurstoftoevoer naar de baby te verhogen, en zo te zorgen dat de baby weer voldoende zuurstof krijgt. Als er een grote verdenking bestaat op zuurstofgebrek, kiezen we er soms voor de baby snel geboren te laten worden, middels een keizersnede of zuignapverlossing.

Voorbeelden van standaard behandelingen om de zuurstoftoevoer naar de baby te verhogen zijn onder andere u op een andere zij draaien, weeënstimulerende medicijnen stop zetten (mocht u die krijgen toegediend), even stoppen met persen of weeënremmende medicijnen toedienen.

In Amerika wordt als standaard behandeling nog iets anders gedaan, namelijk zorgen dat moeder extra zuurstof inademt. De extra zuurstof die de moeder inademt via een kapje op de neus en mond, gaat via de moederkoek naar het bloed van de baby, waardoor het hartslagpatroon zich kan herstellen en waarschijnlijk ook de conditie van de baby verbetert. In Nederland is dit nog geen standaard behandeling.

**Wat is het doel van dit onderzoek?**

Door middel van dit onderzoek willen wij onderzoeken of het toedienen van extra zuurstof aan de moeder zorgt voor minder zuurstofnood bij de baby tijdens het laatste deel van de bevalling. Met dit onderzoek willen we een aantal dingen onderzoeken. Allereerst willen we bekijken of het hartslagpatroon van de baby sneller weer normaal wordt wanneer ook extra zuurstof wordt toegediend, in vergelijking met de standaard behandeling. Daarnaast bepalen we vlak na de geboorte waardes in het bloed die iets zeggen over het zuurstofgehalte van de baby vlak voor de geboorte. Dit is een standaard afname uit de navelstreng die bij alle bevallingen plaatsvindt. Ook willen we in navelstrengbloed nog een aantal extra waardes bepalen om te bepalen hoeveel ‘stress’ de baby heeft gehad tijdens de bevalling. Tenslotte zijn we benieuwd naar hoe u deelname aan dit onderzoek ervaren heeft.

**Wat houdt deelname aan het onderzoek in?**

Het onderzoek begint als er tijdens het persen veranderingen in het hartslagpatroon van de baby optreden die mogelijk kunnen wijzen zuurstofnood. Er wordt dan door een computerprogramma bepaald door middel van loting of u de behandeling met extra zuurstof zult krijgen, of dat u valt in de zogenaamde controlegroep. Als u valt in de controlegroep betekent dit dat u de standaard behandeling krijgt, die kan bestaan uit de boven beschreven behandelingen. Als u extra zuurstof krijgt toegediend gebeurd dit via een maskertje dat over uw neus en mond wordt geplaatst. U moet door dit maskertje ademhalen en zo 100% zuurstof inademen. De verpleegkundige zal u hierbij helpen. Deze behandeling gaat door totdat de baby geboren is. Als deze behandeling niet voldoende is om het hartslagpatroon van de baby te laten herstellen krijgt u hiernaast ook de standaard behandeling. U loopt dus niet het risico dat zuurstofnood bij de baby onbehandeld blijft. Uw behandelend arts of verloskundige zal bepalen welke andere behandelingen nodig zijn om de conditie van de baby zo goed mogelijk te houden. Mocht het hartslagpatroon ondanks alle behandelingen onvoldoende herstellen, dan zal de arts net zoals anders alsnog de baby snel geboren laten worden middels keizersnede of een zuignapverlossing.

Na de bevalling wordt bloed afgenomen uit de navelstreng. In dit bloed bepalen we een aantal waarden, die iets zeggen over de zuurstofvoorraad van de baby vlak voor de geboorte. Dit is standaard bij alle bevallingen en zowel u als de baby merken hier niets van. Voor dit onderzoek willen we 2 buisjes extra bloed afnemen uit de navelstreng waardoor we een aantal extra stofjes kunnen onderzoeken die aangeven hoeveel ‘stress’ de baby heeft ervaren tijdens de bevalling. Ook hiervoor geldt dat zowel u als de baby hier niets van merken.

Uiteraard krijgt u bedenktijd voordat u beslist deel te nemen aan het onderzoek. Zie voor aanvullende informatie over het beslissen om wel of niet deel te nemen de bijgevoegde brochure ‘Medisch wetenschappelijk onderzoek: algemene informatie voor de proefpersoon’. U moet uiterlijk op het moment dat de uitdrijving begint beslissen of u wel of niet deelneemt. Het kost u geen extra tijd en u of de baby hoeven na de bevalling niet vaker dan normaal terug te komen naar het ziekenhuis voor controles.

Wel vragen wij u na de bevalling een korte vragenlijst met 12 meerkeuzevragen in te vullen, om aan te geven hoe u deelname aan het onderzoek heeft ervaren.

**Wat zijn de voordelen van deelname aan dit onderzoek**?

Eerdere onderzoeken maken het aannemelijk dat het zuurstofgehalte van de baby stijgt en hierdoor ook het hartslagpatroon van uw baby verbetert als u extra zuurstof krijgt toegediend tijdens de bevalling. Wij willen dit bevestigen in een onderzoek van betere kwaliteit, onder andere door het toevoegen van een controlegroep. Het zou kunnen zijn dat wanneer het hartslagpatroon beter wordt, de kans dat de arts beslist dat de baby snel geboren moet worden (bijvoorbeeld met behulp van een zuignapverlossing of keizersnede) kleiner wordt. Omdat een zuignapverlossing of keizersnede ook risico’s kennen is zou dit gunstig zijn voor zowel u als de baby.

Behalve de voordelen voor u is het erg belangrijk voor andere zwangeren die in de toekomst zullen bevallen. Wij zullen de resultaten van dit onderzoek openbaar maken. Mocht het zo zijn dat zuurstoftoediening inderdaad de conditie van de baby verbetert tijdens de bevalling, dan zouden andere zwangeren en hun baby’s hier in de toekomst ook van kunnen profiteren. Hierdoor kan de geboortezorg in Nederland worden verbeterd.

**Zijn er risico’s?**

Wij verwachten geen grote risico’s voor u of uw baby door deelname aan dit onderzoek. Bij toediening van 100% zuurstof aan gezonde volwassenen voor andere medische doeleinden (bijvoorbeeld bij de behandeling van ernstige hoofdpijn) zijn geen ernstige bijwerkingen vastgesteld. Ten aanzien van uw baby is bekend dat het aantal vrije zuurstof radicalen in het bloed van de baby stijgt na toediening van zuurstof bij de moeder. Dit zijn stofjes die gedeeltelijk nodig zijn voor ‘normale’ processen in het lichaam, maar als deze in hoge mate aanwezig zijn ook schade kunnen aanrichten. Het aantal van deze stofjes stijgt ook als er sprake is van zuurstofnood, onafhankelijk van of er wel of geen extra zuurstof aan moeder wordt toegediend. Wij verwachten dat de toename van deze stofjes geen schade bij uw kind zal veroorzaken. In Amerika krijgen jaarlijks miljoenen moeders extra zuurstof toegediend tijdens de bevalling zonder dat dit voor problemen bij de pasgeboren baby zorgt.

**Wat is anders dan wanneer u niet zou deelnemen?**

Er zijn twee dingen anders dan de ‘standaard behandeling’ wanneer u deelneemt aan dit onderzoek. Als het computerprogramma bepaalt dat u in de groep valt die extra zuurstof krijgt toegediend, is dit dus anders dan de standaard behandeling in Nederland. Het kan ook zijn dat u deel uitmaakt van de controlegroep, dit betekent dat de behandeling hetzelfde is als wanneer u niet zou deelnemen aan het onderzoek. Onafhankelijk van in welke groep u valt zullen we na de bevalling 2 extra buisjes bloed uit de navelstreng afnemen.

**Wat gebeurt er als u niet wenst deel te nemen aan dit onderzoek?**

Als u niet wenst deel te nemen aan dit onderzoek, verandert er verder niets aan de standaard medische behandeling die u krijgt tijdens de bevalling. Deelname aan dit onderzoek is namelijk geheel op vrijwillige basis. U kunt zich op elk moment uit het onderzoek terugtrekken, ook al hebt u uw medewerking aan het onderzoek al toegezegd. Dit heeft geen gevolgen voor uw verdere medische behandeling. U bent niet verplicht om de reden voor terugtrekking te vertellen.

**Bent u verzekerd wanneer u aan het onderzoek meedoet?**

Er is een risicoverzekering bij MediRisk afgesloten voor patiënten die meedoen aan dit onderzoek.

**Wat gebeurt er met uw gegevens?**

Er wordt vertrouwelijk met uw gegevens omgegaan. De gegevens die in het kader van dit onderzoek worden verzameld, zijn alleen inzichtelijk voor hieronder genoemde onderzoekers. Wij houden ons hierbij aan de Wet Bescherming Persoonsgegevens. Wij zijn verplicht uw onderzoeksgegevens 15 jaar te bewaren. Daarvoor geeft u toestemming als u meedoet aan dit onderzoek.

**Wordt uw huisarts en/of behandelend specialist geïnformeerd bij deelname?**

Nee, omdat we geen nadelen van de behandeling verwachten voor uw algemene gezondheid zal uw huisarts en/of behandelend specialist niet worden geïnformeerd bij deelname aan dit onderzoek. De gynaecologen, gynaecologen in opleiding en verloskundigen werkzaam bij Màxima Medisch Centrum weten wel van deelname aan uw onderzoek.

**Zijn er extra kosten/is er een vergoeding als u mee doet aan dit onderzoek?**

Nee, u krijgt geen vergoeding voor deelname aan dit onderzoek. Dit onderzoek vindt geheel op vrijwillige basis plaats.

**Door wie is dit onderzoek goedgekeurd?**

De Centrale Commissie Mensgebonden Onderzoek en het Ministerie van Volksgezondheid, Welzijn en Sport hebben toestemming gegeven voor het uitvoeren van dit onderzoek. Wat dit betekent, vindt u terug in bijgevoegde brochure ‘Medisch wetenschappelijk onderzoek: algemene informatie voor de proefpersoon’.

**Wilt u verder nog iets weten?**

Indien u na het lezen van deze informatie nog vragen heeft, kunt u hiervoor terecht bij één van de onderstaande onderzoekers. Ook is er altijd een onafhankelijk deskundige beschikbaar voor het beantwoorden van vragen met betrekking tot dit onderzoek. Dit is een arts die zelf niet betrokken is bij het onderzoek, maar hier wel veel vanaf weet. De gegevens van deze arts staan hieronder genoemd.

**Wat kunt u doen als u een klacht heeft?**

Als u niet tevreden bent over het onderzoek, kunt u terecht bij de onafhankelijke klachtencommissie van het Máxima Medisch Centrum. De klachtencommissie is te bereiken op telefoonnummer: 040-888 9115.

**Onderzoekers betrokken bij dit project**

Drs. Lauren Bullens, arts in opleiding tot gynaecoloog, Maastricht Universitair Medisch Centrum

Drs. Olenka Hulsenboom, arts-onderzoeker, Máxima Medisch Centrum

Dr. Ir. Beatrijs van der Hout-van der Jagt, ingenieur-onderzoeker, Máxima Medisch Centrum

Dr. Pieter van Runnard Heimel, gynaecoloog-perinatoloog, Máxima Medisch Centrum

Prof. Dr. Guid Oei, gynaecoloog-perinatoloog, Máxima Medisch Centrum

Telefoon: 040-888 9551 (verloskamers)

Email: [laurenbullens@gmail.com](mailto:laurenbullens@gmail.com)

**Onafhankelijk onderzoeker**

Dr. Peter Andriessen, kinderarts-neonatoloog

Email: p.andriessen@mmc.nl

Telefoon: 040-888 9350

**Bijlagen**

- Schriftelijke verklaring van toestemming

- Brochure ‘Medisch wetenschappelijk onderzoek: algemene informatie voor de proefpersoon’

NL53018.000.15 Toestemmingsformulier (kopie voor proefpersoon)

**Schriftelijke verklaring van toestemming**

*Kan zuurstofnood van het ongeboren kind tijdens de bevalling worden behandeld door het toedienen van extra zuurstof aan de moeder?*

*(INTEREST-O2)*

Ik heb de informatiebrief voor de proefpersoon gelezen. Ik kon aanvullende vragen stellen. Mijn vragen zijn voldoende beantwoord. Ik had genoeg tijd om te beslissen of ik meedoe. Ik weet dat meedoen helemaal vrijwillig is. Ik weet dat ik op ieder moment kan beslissen om alsnog niet mee te doen. Daarvoor hoef ik geen reden te geven.

Ik weet dat de onderzoekers mijn gegevens kunnen zien. Ik geef toestemming om mijn gegevens te gebruiken, voor de doelen die in de informatiebrief staan. Ik geef toestemming om mijn onderzoeksgegevens 15 jaar na afloop van dit onderzoek te bewaren.

Ik wil meedoen aan dit onderzoek.

Naam proefpersoon:

Handtekening: Datum : __ / __ / __

-----------------------------------------------------------------------------------------------------------------

Ik verklaar hierbij dat ik deze proefpersoon volledig heb geïnformeerd over het genoemde onderzoek. Als er tijdens het onderzoek informatie bekend wordt die de toestemming van de proefpersoon zou kunnen beïnvloeden, dan breng ik haar daarvan tijdig op de hoogte.

Naam onderzoeker (of diens vertegenwoordiger):

Handtekening: Datum: __ / __ / __

-----------------------------------------------------------------------------------------------------------------

Aanvullende informatie is gegeven door (indien van toepassing):

Naam:

Functie:

Handtekening: Datum: __ / __ / __

NL53018.00.15 Toestemmingsformulier (kopie voor onderzoeksdossier)

**Schriftelijke verklaring van toestemming**

*Kan zuurstofnood van het ongeboren kind tijdens de bevalling worden behandeld door het toedienen van extra zuurstof aan de moeder?*

*(INTEREST-O2)*

Ik heb de informatiebrief voor de proefpersoon gelezen. Ik kon aanvullende vragen stellen. Mijn vragen zijn voldoende beantwoord. Ik had genoeg tijd om te beslissen of ik meedoe. Ik weet dat meedoen helemaal vrijwillig is. Ik weet dat ik op ieder moment kan beslissen om alsnog niet mee te doen. Daarvoor hoef ik geen reden te geven.

Ik weet dat de onderzoekers mijn gegevens kunnen zien. Ik geef toestemming om mijn gegevens te gebruiken, voor de doelen die in de informatiebrief staan. Ik geef toestemming om mijn onderzoeksgegevens 15 jaar na afloop van dit onderzoek te bewaren.

Ik wil meedoen aan dit onderzoek.

Naam proefpersoon:

Handtekening: Datum : __ / __ / __

-----------------------------------------------------------------------------------------------------------------

Ik verklaar hierbij dat ik deze proefpersoon volledig heb geïnformeerd over het genoemde onderzoek. Als er tijdens het onderzoek informatie bekend wordt die de toestemming van de proefpersoon zou kunnen beïnvloeden, dan breng ik haar daarvan tijdig op de hoogte.

Naam onderzoeker (of diens vertegenwoordiger):

Handtekening: Datum: __ / __ / __

-----------------------------------------------------------------------------------------------------------------

Aanvullende informatie is gegeven door (indien van toepassing):

Naam:

Functie:

Handtekening: Datum: __ / __ / __
